# Morphometry and Frequency of the Pyramidalis Muscle in Adult Humans: A Pyramidalis Muscle’s Anatomical Analysis

**DOI:** 10.6061/clinics/2020/e1623

**Published:** 2020-07-06

**Authors:** Flávio Carneiro Hojaij, Rudolph Octaviano Kogima, Raquel Ajub Moyses, Flávia Emi Akamatsu, Alfredo Luiz Jacomo

**Affiliations:** Departamento de Cirurgia, Faculdade de Medicina FMUSP, Universidade de Sao Paulo, Sao Paulo, SP, BR

**Keywords:** Anatomical Variation, Corpse, Frequency Measurements, Pyramidalis Muscle

## Abstract

**OBJECTIVES:**

To verify the pyramidalis muscle’s frequency (bilaterality, unilaterality, or absence) and morphometry (length of the medial border and width of its origin/base) in a sample of the Brazilian population and the anthropometric influence.

**METHODS:**

Dissection of 30 cadavers, up to 24h post-mortem.

**RESULTS:**

The pyramidalis muscle was present bilaterally and unilaterally in 83.33% and 3.33% of the cadavers, respectively, and absent in 13.33%. The muscles on the right and left sides were symmetrical in length but not in width; the pyramidalis muscles of men were longer, while those of the women were wider. We also found that there was greater variation in the dimensions (length and width) of the men’s muscles. Finally, in this sample of the Brazilian population, the pyramidalis muscle’s unilaterality was more prevalent than in other populations, and its complete absence was less prevalent.

**CONCLUSIONS:**

There were no cases of muscle duplication in one or both sides, as described in some studies. Despite all of its morphometric variation, the pyramidalis muscle maintained its triangular shape with longitudinal fibers in every case. Furthermore, no statistically significant correlation was noted between the muscles’ dimensions and person’s age, height, weight, or gender.

## INTRODUCTION

The pyramidalis muscle is a small triangular muscle located in the inferior part of the anterior abdominal wall (1). It originates on the pubis’ anterior-superior surface, upon which it is inserted by the tendinous fibers that in the pubic symphysis region may merge with the suspensory ligament of the penis. The pyramidalis, then, extends upward, narrowing until it reaches its insertion place, the linea alba, midway between the umbilicus and the pubic symphysis. Regarding the stratigraphy, the belly of the pyramidalis muscle is located between the rectus abdominis and rectus sheath. Its morphometry is variable: the length of its medial border usually ranges from 2 to 13.8 cm; the width of its base measures an average of 1.98 cm (2). There is no relation between the muscle’s length and person’s height (3). The pyramidalis muscle’s innervation always comes from the anterior cutaneous branches of the intercostal nerves, but there is considerable variation in the spinal nerves from which this innervation originates, involving Th12, L1, and L2 (4). The pyramidalis muscle does not present pennation: its fibers extend vertically (5). It is not a constant muscle since it can be bilateral, unilateral, duplicated in one or both sides (presenting, respectively, three and four bellies), or even absent (6). The absence of the pyramidalis muscle is rare, but it has been preponderant in some studies (7). Finally, the pyramidalis seems to be more constant in African and South East populations than in white ones (8).

It is believed that the pyramidalis muscle’s function is to strengthen the abdominal wall (9) and tense the linea alba. However, its absence does not cause apparent loss of function (2). Hence, despite the aforementioned extensive description, the pyramidalis muscle’s function is still unclear. Because of that, some authors consider it a vestigial muscle, leftover from the marsupials’ and monotremes’ pouch (10), evolving into an irregular apparition in more evolved primates, often observed in chimpanzees and gorillas but absent in orangutans (11). There are also anthropological studies suggesting that the pyramidalis has evolved as part of the normal human anatomy, in which case it could be related to the species’ erect posture.

Although its function is not well known, the pyramidalis muscle can be useful in some surgical procedures since its superior insertion may serve as reference for abdominal incisions, for example, Pfannenstiel (10). Furthermore, given that, as mentioned earlier, its absence does not seem to cause notable dysfunction (2), it can be used for grafts. Cryopreserved, the pyramidalis muscle can be used as a source of stem cells, which in turn may be used in the treatment of post‐prostatectomy stress urinary incontinence (12). However, there are still few publications describing the muscle. The most recently published articles have discussed its morphometry in Greece (13), India (14), and North India (6).

Considering this literary scarceness, the inexistence of morphometric data for the muscle in Brazil, and the potential surgical applications of this data, collecting it becomes considerably relevant to verify if, indeed, the pyramidalis muscle could be a good incision reference in the Brazilian population and add to the literature regarding this muscle. Therefore, this study verifies the pyramidalis muscle’s frequency (including unilaterality, bilaterality, absence, and other anatomical variations) in a sample of the Brazilian population and describes its morphometry (length of its medial border and width of its base). The results allowed comparisons between genders and with studies of other populations.

## MATERIALS AND METHODS

**Figure 1:**
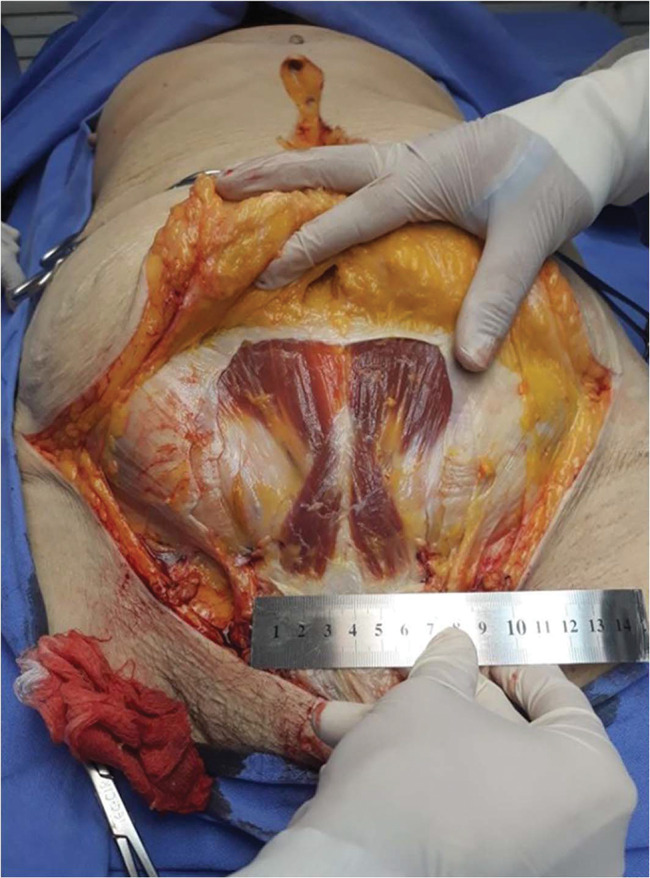
Dissection methods and measurements. Global vision of the inferior abdominal wall dissected according to this study’s methods, showing a pyramidalis muscle with average dimensions.

**Figure 2:**
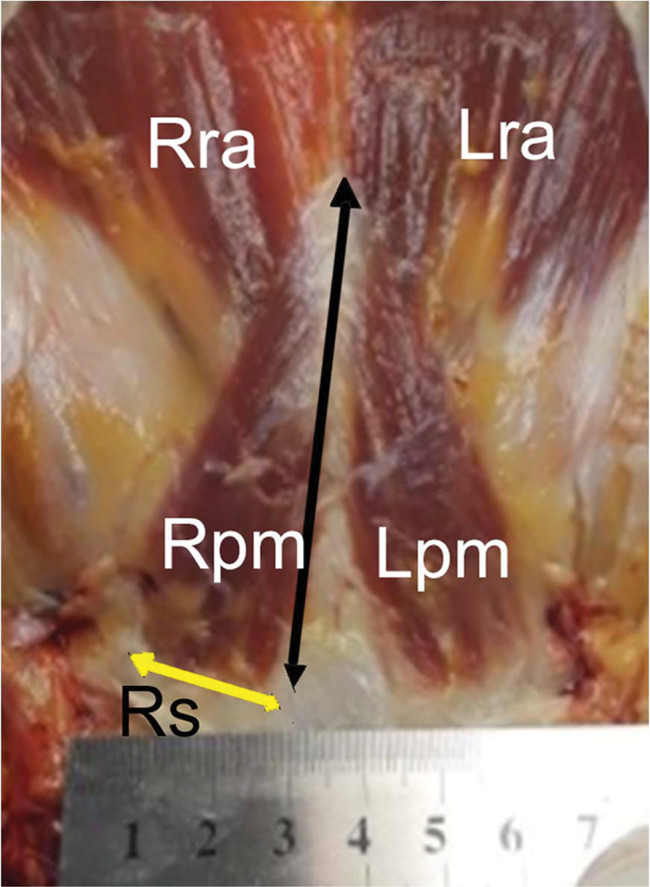
Dissection methods and measurements. Zoom window of the same dissection of Figure 1 (cadaver positioned with the head upward and feet downward), showing the right and left pyramidalis muscles (Rpm and Lpm, respectively), right and left rectus abdominis (Rra and Lra, respectively) underneath the pyramidalis, and part of the rectus sheath (Rs), cut and folded, exposing these muscles. The longer arrow indicates the pyramidalis muscle’s medial border, where the length was measured; the shorter arrow indicates its base, where the width was measured.

The study comprised 30 fresh cadavers (11 females and 19 males) of individuals from 32 to 98 years old, provided by the Serviço de Verificação de =bitos da Capital da Universidade de São Paulo (SVOC/USP). Regarding their ethnicity, 27 were white and three were nonwhite. Cadavers that had been submitted to previous incisions in the abdomen’s inferior region, which could alter the pyramidalis muscle’s morphology, were excluded from the study and not computed among the 30. Each approved cadaver was dissected during the medical residents’ dissection class; no procedures were performed outside of these class periods. The dissections were made through surgical incisions and performed according to the following procedures:

- Median longitudinal incision in the suprapubic and infraumbilical regions and two symmetrical bilateral incisions (Pfannenstiel-like). These three incisions were made through the skin and subcutaneous cellular tissue, forming flaps that preserved the superficial layer of the rectus sheath. Afterward, two parallel longitudinal incisions were made in the lateral borders of the rectus abdominis, going through the anterior layer of the rectus sheath. Finally, one transverse incision was made connecting those longitudinal incisions so that the aponeurosis flap could be folded, exposing both the rectus abdominis and median line and allowing the study of the pyramidalis muscles (if present) (Figure 1).
- Exposure of the pyramidalis muscles without releasing them from their original place, allowing for photographic recording for posterior measuring (length of the medial border and width of the origin/base) in image processing program ImageJ. During the dissection, there was no removal of the structures from the cadavers (Figure 2).
- Suture by layers, according to the SVOC/USP routine, after the collection of the data, which was processed and then compared with data from other studies.

The study was approved by the “Plataforma Brasil” (Brazilian Platform), according to the Brazilian Ministry of Health, National Health Council, and National Commission for Research Ethics (CONEP-Comiss�o Nacional de �tica em Pesquisa) (number 2.178.874, 06/07/2017). Therefore, the present study has been performed with the Declaration of Helsinki’s ethical standards. The dissections were made in accordance with the SVOC/USP regulations, which respect the guidelines related to cadaver studies and dissecting. Informed consent, made by the SVOC/USP, was obtained post-mortem by the next of kin.

Regarding statistical analysis, the frequencies and percentages are shown for the categorical variables. For continuous variables, means and standard deviations were calculated. The Shapiro-Wilk W test was performed to test for normality of continuous data. The two-sample t-test and Wilcoxon/Kruskal-Wallis tests were used for comparison of parametric and nonparametric data, respectively. Linear regressions were performed to test association between continuous data. A two-tailed *p*-value of 0.05 was considered statistically significant. Given the exploratory nature of the study, no adjustment for multiple analyses was performed.

## RESULTS

**Figure 3:**
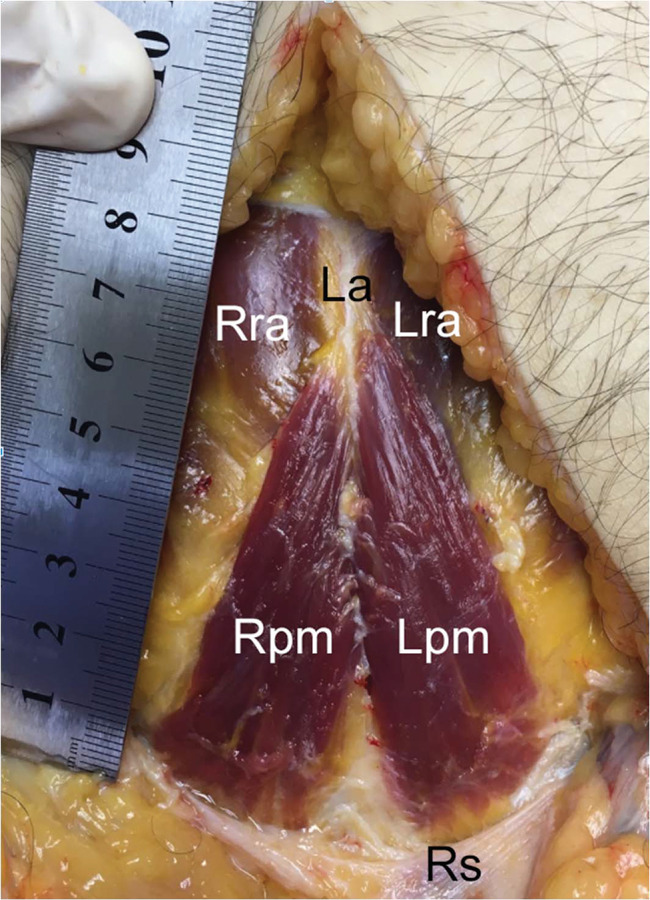
Pyramidalis muscle with average dimensions. Left side: length, 7.73 cm; width, 2.33 cm. Right side: length, 7.23 cm; width, 2.64 cm. Note: cadaver positioned with the head upward and feet downward. Right pyramidalis muscle, Rpm; left pyramidalis muscle, Lpm; right rectus abdominis, RRA; left rectus abdominis, Lra; linea alba, La; rectus sheath, Rs.

**Figure 4:**
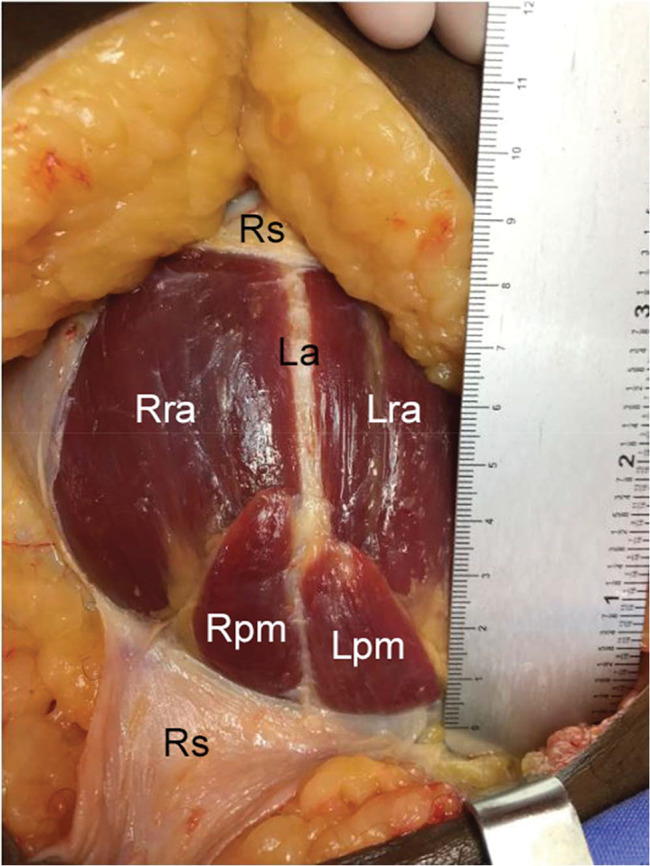
Short and wide pyramidalis muscle. Left side: length, 3.12 cm; width, 2.26 cm. Right side: length, 3.96 cm; width, 1.97 cm. Note: cadaver positioned with the head upward and feet downward. Right pyramidalis muscle, Rpm; left pyramidalis muscle, Lpm; right rectus abdominis, Rra; left rectus abdominis, Lra; linea alba, La; rectus sheath, Rs.

**Figure 5:**
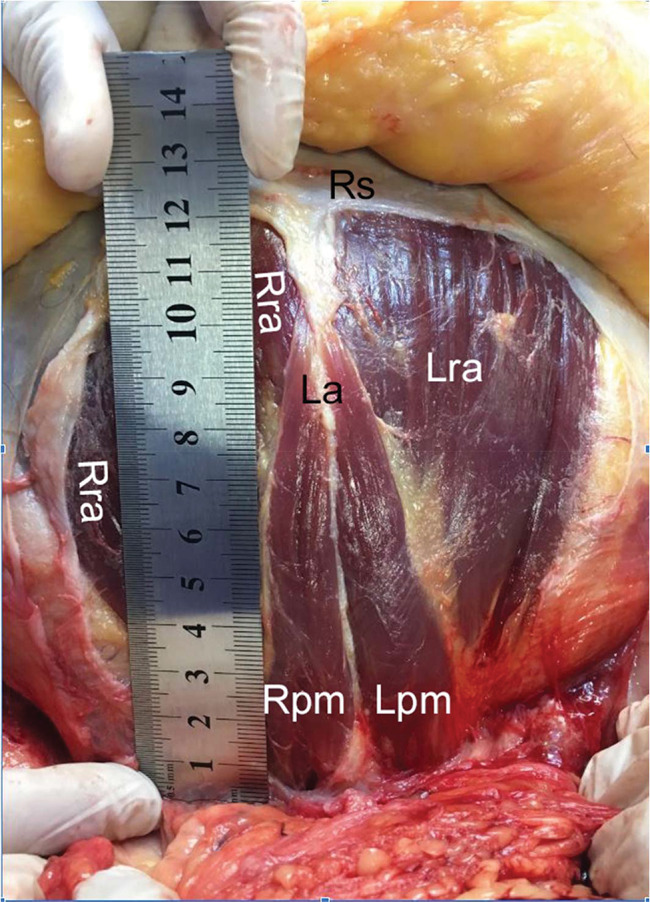
Long pyramidalis muscle. Left side: length, 10.20 cm; width, 1.93 cm. Right side: length, 9.95 cm; width, 1.49 cm. Note: cadaver positioned with the head upward and feet downward. Right pyramidalis muscle, Rpm; left pyramidalis muscle, Lpm; right rectus abdominis, Rra; left rectus abdominis, Lra; linea alba, La; rectus sheath, Rs.

**Figure 6:**
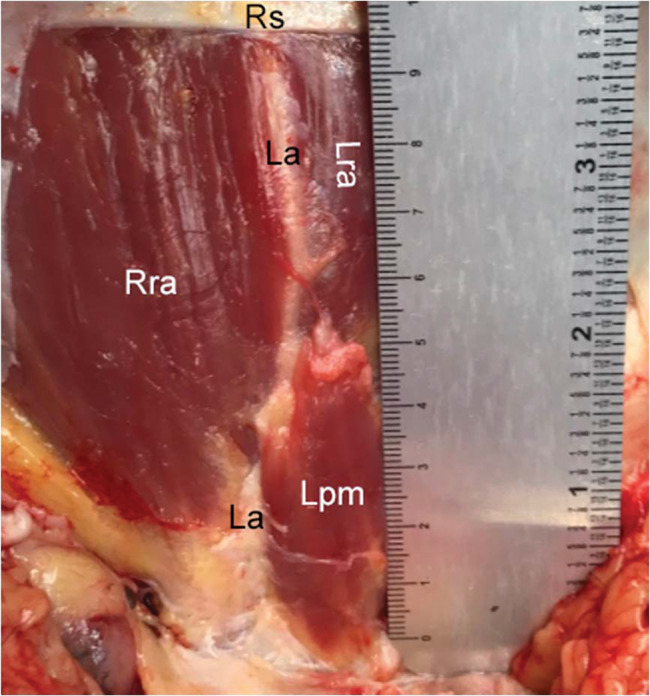
Unilateral left pyramidalis muscle. The linea alba can be seen as a vertical line by the right side of the muscle. Left side: length, 5.70 cm; width, 2.49 cm. Left pyramidalis muscle, Lpm; right rectus abdominis, Rra; left rectus abdominis, Lra; linea alba, La; rectus sheath, Rs.

**Figure 7:**
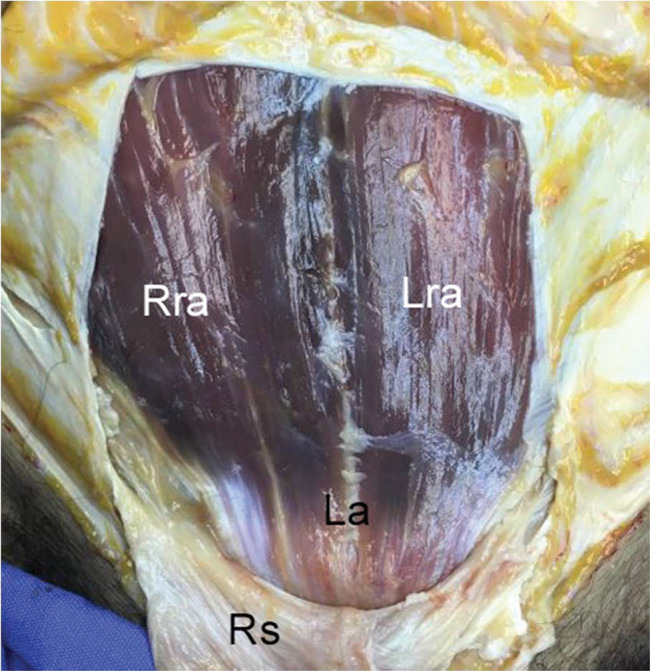
A case of absent pyramidalis muscle. Right rectus abdominis, Rra; left rectus abdominis, Lra; linea alba, La; rectus sheath, Rs.

**Figure 8:**
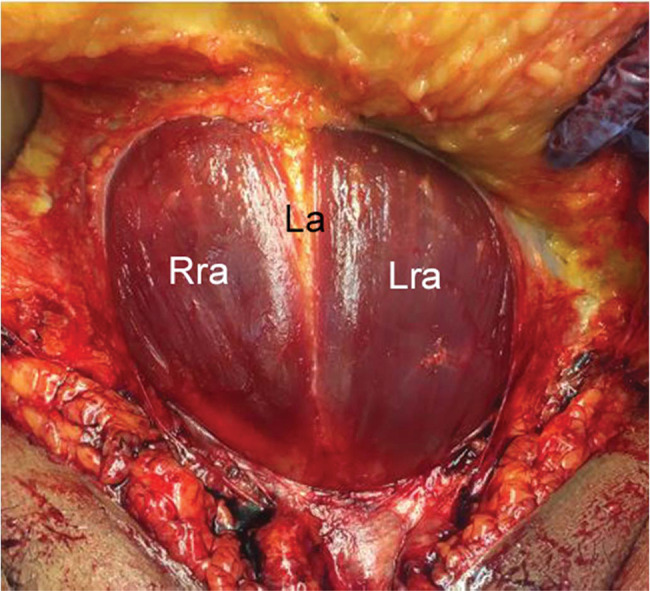
Another case of absent pyramidalis muscle. Right rectus abdominis, Rra; left rectus abdominis, Lra; linea alba, La; rectus sheath, Rs.

The pyramidalis muscle was present bilaterally in 25 out of the 30 cadavers (83.33%; Figures 3, 4, and 5) and unilaterally (left-sided, Figure 6) in 1 (3.33%) and absent in 4 (13.33%, Figures 7 and 8). Regarding its dimensions, the length of its medial border ranged from 3.12 to 10.67 cm on the left side and 3.50 to 10.76 cm on the right side, and the width of its base ranged from 0.91 to 2.93 cm on the left side and 1.10 to 2.64 cm on the right side (Table 1).

The mean length of the medial border on the left side was 6.64±2.04 cm, and that on the right side was 6.80±2.14 cm. The mean width of both the left and right pyramidalis muscles’ bases was 1.87±0.45 cm (Table 1).

Among the 11 women, we found 1 (9.09%) case of pyramidalis absence and no cases of unilaterality; the pyramidalis was present bilaterally in 10 (90.90%) of them. The length of the muscle’s medial border ranged from 3.12 to 9.50 cm on the left side and 3.96 to 9.00 cm on the right side; the width of the pyramidalis’ base ranged from 1.47 to 2.48 cm on the left side and 1.41 to 2.50 cm on the right side (Table 1).

For women, the mean length of the medial border of the left-sided pyramidalis was 6.38±1.93 cm, and that of the right-sided pyramidalis was 6.42±1.78 cm. The mean width of the left pyramidalis’ base was 1.94±0.30 cm, and that of the right pyramidalis’ base was 1.91±0.35 cm (Table 1).

Among the 19 men, the pyramidalis was absent in 3 (15.78%) of the cadavers and present unilaterally in 1 (5.26%) and bilaterally in 15 (78.94%). The length of the muscle’s medial border ranged from 3.50 to 10.67 cm on the left side and 3.50 to 10.76 cm on the right side; the width of the pyramidalis’ base ranged from 0.91 to 2.93 cm on the left side and 1.10 to 2.64 cm on the right side (Table 1).

For men, the mean length of the medial border of the left-sided pyramidalis was 6.80±2.16 cm, and that of the right-sided pyramidalis was 7.06±2.38 cm. The mean width of the left pyramidalis’ base was 1.83±0.53 cm, and that of the right pyramidalis’ base was 1.85±0.52 cm (Table 1).

**Figure 9:**
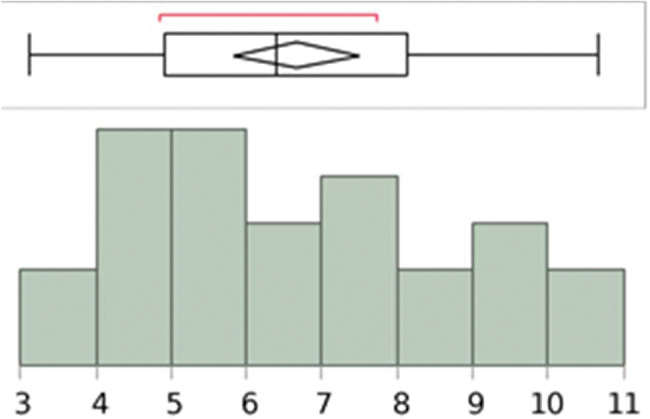
Analysis with the Shapiro–Wilk W test to show the normal distribution of the measurements (cm) of the pyramidalis muscles’ length at the medial border of the left side. W=0.975129; *p*-value=0.7578. Note: Ho=the data is from the normal distribution. Small *p*-values reject Ho.

**Figure 10:**
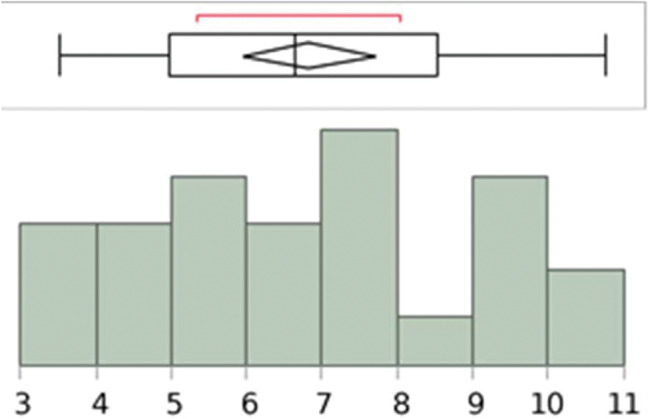
Analysis with the Shapiro–Wilk W test to show the normal distribution of the measurements (cm) of the pyramidalis muscles’ length at the medial border of the right side. W=0.959641; *p*-value=0.4074. Note: Ho=the data is from the normal distribution. Small *p*-values reject Ho.

**Figure 11:**
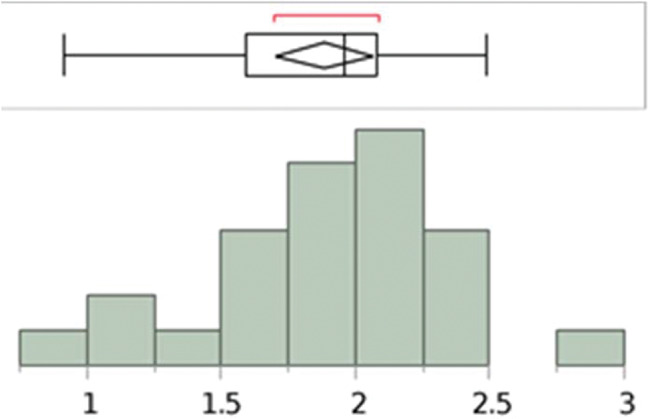
Analysis with the Shapiro–Wilk W test to show the normal distribution of the measurements (cm) of the pyramidalis muscles’ width at the base of the left side. W=0.964588; *p*-value=0.4898. Note: Ho=the data is from the normal distribution. Small *p*-values reject Ho.

**Figure 12:**
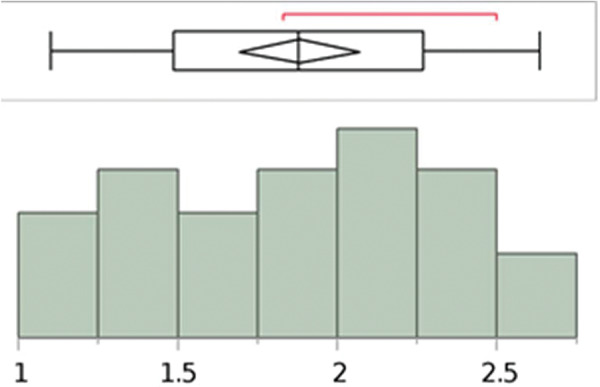
Analysis with the Shapiro–Wilk W test to show the normal distribution of the measurements (cm) of the pyramidalis muscles’ width at the base of the right side. W=0.961571; *p*-value=0.4466. Note: Ho=the data is from the normal distribution. Small *p*-values reject Ho.

The length and width measurements of the pyramidalis muscle, on both right and left sides, showed a normal distribution (Figures 9, 10, 11, and 12).

**Figure 13:**
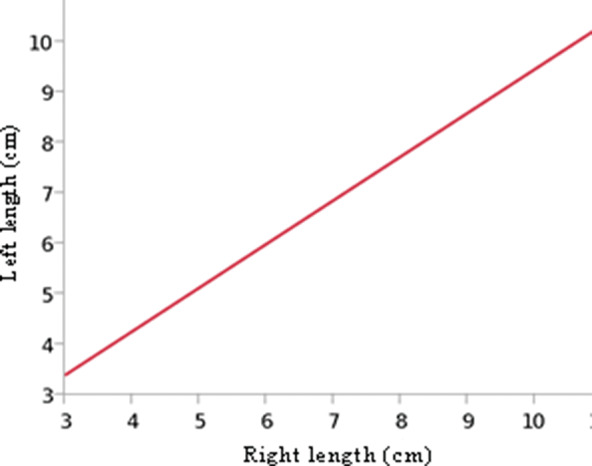
Linear regression showing symmetry between the right and left sides of the pyramidalis muscle’s length (cm) at its medial border. Linear fit given as follows: left length (cm)=0.7878651+0.8658011*right length (cm); F ratio=90.6430; *p*-value<0.0001.

**Figure 14:**
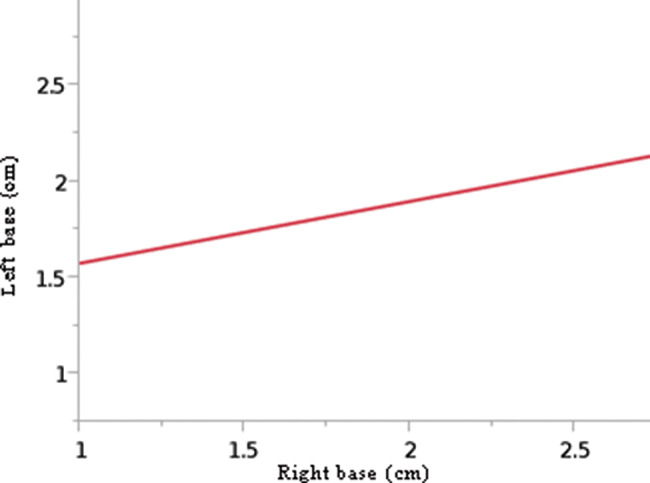
Linear regression showing asymmetry between the right and left sides of the pyramidalis muscle’s width (cm) at its base. Linear fit given as follows: left base (cm)=1.2503428+0.3213754*right base (cm). F ratio=2.7603; *p*-value=0.1102.

Through linear regression, we found that the pyramidalis muscle’s length was symmetrical (RSquare: 0.79; *p*<.0001) but the width at its base was not (RSquare: 0.10; p=0.1102). In other words, if the pyramidalis muscle is long or short on one side, the contralateral muscle tends to have the same length. However, conversely, whether the pyramidalis muscle is wide or narrow on one side, nothing can be predicted about the other side’s width (Figures 13 and 14). It must be emphasized that despite the statistical length symmetry, there were exceptional cases with considerable length asymmetry. For example, in one case, the medial border of the pyramidalis measured 6.47 cm on the left side and 10.76 cm on the right side (Figure 7).

In the present study, we can also see that the range of the dimensions (length and width) of the pyramidalis muscle was bigger in men.

The mean length of the muscle was bigger in men than in women: the mean length on the left side was 6.80±2.16 cm in men and 6.38±1.93 cm in women, that on the right side was 7.06±2.38 cm in men and 6.42±1.78 cm in women, and the combined mean length (considering the right and left sides altogether) was 6.92±2.23 cm in men and 6.40±1.81 cm in women (Table 1).

Women had wider pyramidalis muscles: the mean width of the left pyramidalis’ base was 1.94±0.30 cm in women and 1.83±0.53 cm in men, that of the right pyramidalis’ base was 1.91±0.35 cm in women and 1.85±0.52 cm in men, and the combined mean width was 1.92±0.31 cm in women and 1.84±0.52 cm in men (Table 1).

**Figure 15:**
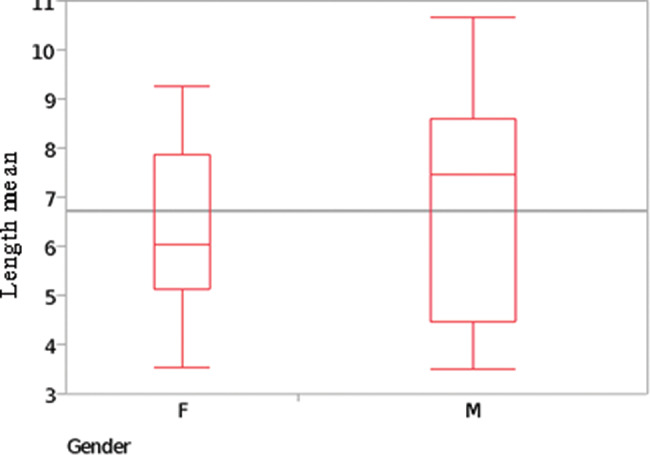
Correlation analysis between gender and the pyramidalis muscle’s mean length (cm) at its medial border. Females, F; males, M. Sample test, normal approximation: S=120; Z=-0.52697; *p*-value=0.5982.

**Figure 16:**
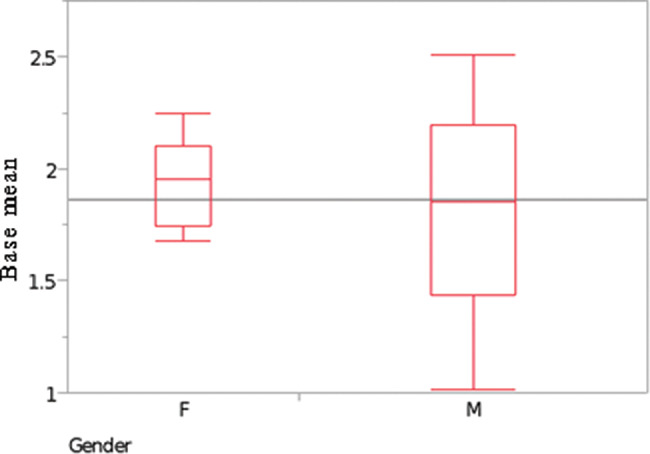
Correlation analysis between gender and the pyramidalis muscle’s mean width (cm) at its base. Females, F; males, M. Sample test, normal approximation: S=139; Z=0.47150; *p*-value=0.6373.

Statistically, however, this difference regarding length and width in genders was not significant (*p*=0.5982 and *p*=0.6373, respectively) (Figures 15 and 16).

Linear regression was also used to analyze correlation between mean length and height, mean width and height, mean length and weight, mean width and weight, mean length and age, and mean width and age. None of these analyses showed any correlation (Table 2).

## DISCUSSION

Comparing our data with studies on other populations, the Brazilian population had an average incidence of the pyramidalis muscle (Table 3). In Table 3, we can see a considerable range of incidences, from 72% in the Indian population up to 99% in the Chinese; in this study, the Brazilian population’s sample presented an incidence of 83.33%.

In this sample of the Brazilian population, the unilaterality of the pyramidalis was less prevalent than in other populations, and its absence was more prevalent. Both Brazilian men and women presented wider pyramidalis than other populations (Table 4).

**Figure 17:**
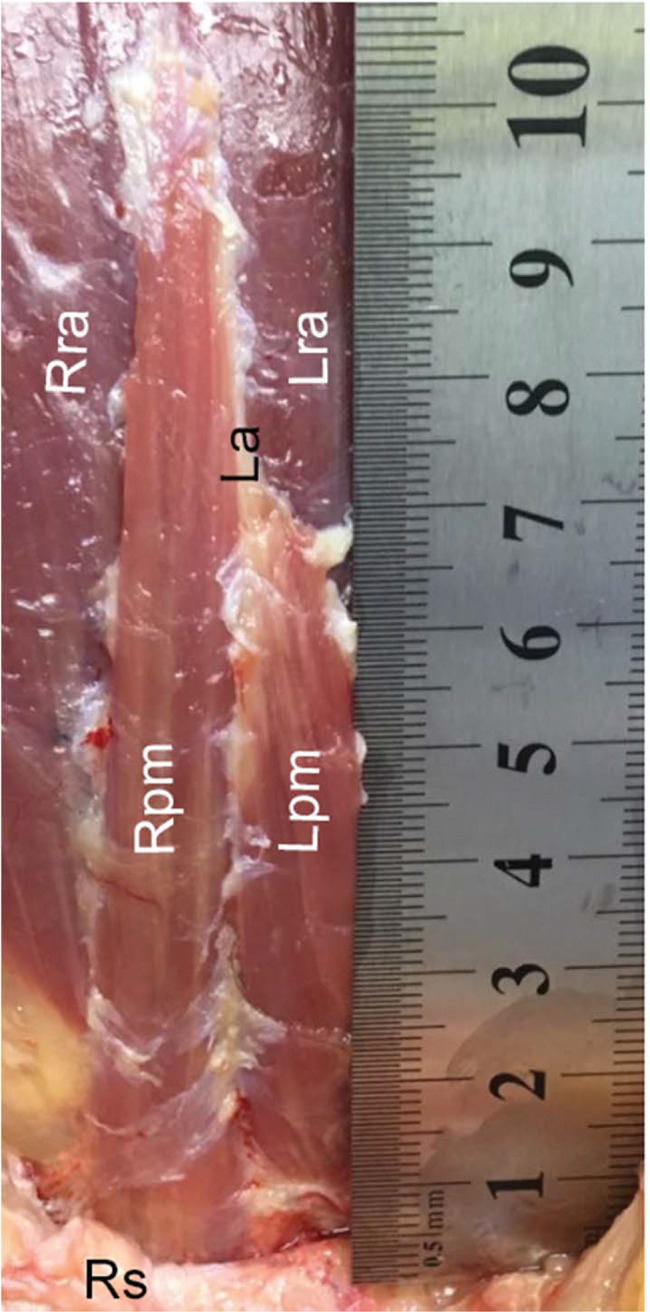
Anatomical variations. Asymmetrical pyramidalis muscle. Left side: length, 6.47 cm; width, 1.53 cm. Right side: length, 10.76 cm; width, 1.24 cm.

**Figure 18:**
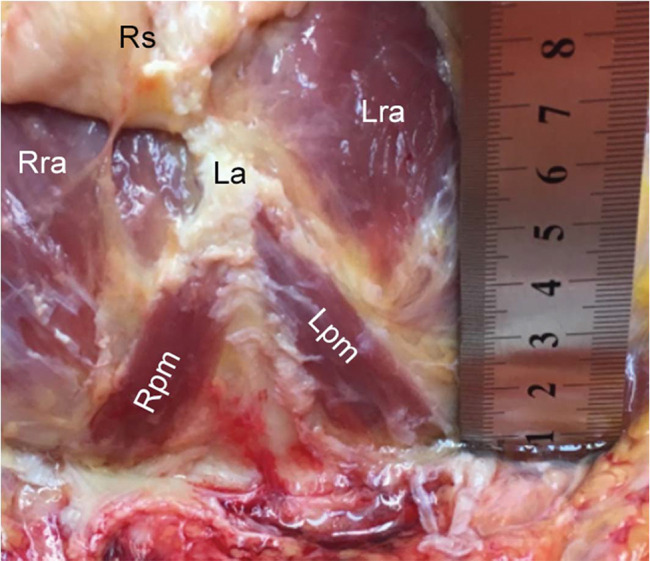
Anatomical variations. Contralateral pyramidalis muscles widely separated from each other at the base. Left side: length, 5.34 cm; width, 1.76 cm. Right side: length, 5.34 cm; width, 1.68 cm.

Despite all statistical data and descriptions collated, the pyramidalis muscle’s great variability on morphometry and frequency (Figures 17 and 18), found in this and other studies, makes it hard to predict what will be found when incising a person’s abdomen.

In this study, we found that the pyramidalis muscle’s dimensions have no statistical correlations to age, gender, height, or weight (at least in this sample of the Brazilian population). Therefore, considering the clinical applications of our findings, it seems to be a poor reference for incisions because of its inconsistency.

In contrast, the pyramidalis proved to be a very prevalent muscle, present in more than 70% of the people in populations from other studies and 83.33% of the corpses from this study (Table 3). This increases its viability as a graft since, in spite of its high prevalence, its absence does not seem to cause any dysfunction. Therefore, supposedly, it can be removed and used for grafts with no collateral effects. Moreover, the mean length and width of the pyramidalis muscle in this study provide an estimate of the size of the lesions that can be treated with the pyramidalis muscles’ grafts in the Brazilian population.

Furthermore, clearly, the high prevalence of the muscle in the Brazilian population may enable its use as a source of stem cells although this possibility is still being studied.

Finally, we can say that the pyramidalis muscle is still a great mystery for science: the variability in its frequency and morphometry (in this and other studies) and indifference of its absence perpetuate the uncertainties about its functions. Its phylogenetic origin has not yet been truly elucidated, which makes it difficult to ascertain its future development: while some studies say that the pyramidalis muscle is a vestigial muscle (thus tending to disappear in the future), others argue that its presence is related to the erect posture (thus tending to become more prevalent).

## CONCLUSION

In this sample of the Brazilian population, the pyramidalis muscle was present bilaterally in 83.33% of the cadavers and unilaterally in 3.33% and absent in 13.33%. There were no cases of duplication of the muscle in one or both sides, as described in some studies.

In some cases, there was great asymmetry in both length and width. However, statistically, it was found that there was length symmetry between the left and right pyramidalis muscles, but no width symmetry. Despite all of its morphometric variation, the pyramidalis muscle maintained its triangular shape with longitudinal fibers in every case.

## AUTHOR CONTRIBUTIONS

Hojaij FC conceived the research theme and dissection method, supervised the dissections, and was responsible for the manuscript writing. Kogima RO conducted the dissections, made the computer measurements, and wrote the manuscript. Jacomo AL and Akamatsu FE supervised the dissections and conceived the dissection method. Moyses RA made the statistical analysis. All authors reviewed the manuscript.

## Figures and Tables

**Table 1:** Incidence and dimensions of the pyramidalis muscle in the dissected cadavers. Description of the cases (separated in male, female, and total), with the incidence of the pyramidalis muscle (absence, unilaterality, and bilaterality) and maximum, minimum, and mean dimensions (length of the medial border and width of its base).

Incidence and dimension	Side	Male (n=19)	Female (n=11)	Total (n=30)
Absent		15.78%	9.09%	13.33%
Unilateral		5.26%	0%	3.33%
Bilateral		78.94%	90.90%	83.33%
Mean length±sd (cm)	Left	6.80±2.16	6.38±1.93	6.64±2.04
	Right	7.06±2.38	6.42±1.78	6.80±2.14
Combined mean length (cm)		6.92±2.23	6.40±1.81	6.72±2.07
Mean width±sd (cm)	Left	1.83±0.53	1.94±0.30	1.87±0.45
	Right	1.85±0.52	1.91±0.35	1.87±0.45
Combined mean width (cm)		1.84±0.52	1.92±0.31	1.87±0.45
Length range (cm)	Left	3.50-10.67	3.12-9.50	3.12-10.67
	Right	3.50-10.76	3.96-9.00	3.50-10.76
Width range (cm)	Left	0.91-2.93	1.47-2.48	0.91-2.93
	Right	1.10-2.64	1.41-2.50	1.10-2.64

**Table 2:** Analysis of the anthropometric influence (height, weight, and age) upon the pyramidalis muscles’ dimensions (mean length of the medial border and mean width of its base).

Parameters	RSquare	*p*-value
Mean length *vs*. height	0.008639	0.6586
Mean width *vs*. height	0.012315	0.5974
Mean length *vs*. weight	4,72E-02	0.9740
Mean width *vs*. weight	0.04085	0.3326
Mean length *vs*. age	0.000125	0.9577
Mean width *vs*. age	0.054748	0.2603

Note: the influence of ethnicity was not analyzed because of the poor ethnic diversity of the sample.

**Table 3:** Pyramidalis muscle’s incidence in different populations (based on Kaur et al. (6)).

Authors	Population	Incidence
Das (14)	Indian	72%
Loth (11)	African	79%
Vallois (15)	African	82%
Le Double (16)	French	89%
Kaur et al. (6)	North Indian	93.33%
Mori (17)	Japanese	94.5%
Wagenseil (18)	Chinese	99%
Present study	Brazilian	83.33% (25/30)

**Table 4:** Pyramidalis muscle’s incidence of unilaterality, bilaterality, and absence and mean length and width per gender in different populations (based on Das et al. (14)).

						Mean length (cm)		Mean width (cm)	
Authors	Population	Unilateral	Bilateral	Absent	Gender	Right	Left	Right	Left
Didia et al. (19)	Nigerian	-	91.67%	8.33%		8.09	7.94	1.55	1.60
Natsis et al. (13)	Greek	14.60%	79.2%	6.2%	Male	8.37	7.50	1.61	1.56
					Female	6.18	6.56	1.50	1.55
Kaur et al. (6)	North Indian	6.67%	93.33%	-	Male	4.97	4.97	1.75	1.75
					Female	4.87	4.78	1.25	1.45
Das et al. (14)	Indian	20.00%	72.00%	8.00%	Male	5.22	5.39	1.83	1.70
					Female	5.01	5.12	1.78	1.62
Present study	Brazilian	3.33%	83.33%	13.33%	Male	7.06	6.80	1.85	1.83
					Female	6.42	6.38	1.91	1.94
